# Zinc sulfate improves insulin resistance, oxidative stress and apoptosis in liver tissues of PCOS rats through the NF-κB pathway

**DOI:** 10.3389/fendo.2025.1569866

**Published:** 2025-06-06

**Authors:** Yi-fan Kang, Jia-yi Zhao, Jian-rong Liu

**Affiliations:** ^1^ The Fifth Clinical Medical College of Shanxi Medical University, Shanxi Provincial People’s Hospital, Taiyuan, China; ^2^ Department of Reproductive Medicine, Shanxi Provincial People’s Hospital, Taiyuan, China

**Keywords:** zinc sulfate, polycystic ovary syndrome, oxidative stress, mitochondrial damage, hepatic lipid deposition

## Abstract

**Background:**

Polycystic ovary syndrome (PCOS) is primarily characterized by insulin resistance, which leads to increased hepatic glucose production and impaired insulin-mediated glucose disposal. Pathologically, this condition manifests as elevated liver cell apoptosis and reduced lipid transport capacity, further exacerbating insulin resistance. Liver cell apoptosis and mitochondrial dysfunction are key pathological features of PCOS-associated liver diseases, contributing significantly to the progression of PCOS. Although zinc sulfate is recognized for its antioxidant properties, its efficacy in ameliorating PCOS-related liver damage remains unclear.

**Methods:**

Female Sprague-Dawley rats were induced with PCOS and non-alcoholic fatty liver disease (NAFLD) through a high-fat diet and letrozole administration over 28 days. Subsequently, the model rats received zinc sulfate treatment via gavage once daily for an additional 21 days. Serum hormone levels and biochemical markers were assessed using ELISA and enzymatic assays. Histological examination of ovarian and liver tissues was performed using hematoxylin and eosin (HE) staining, while hepatic lipid accumulation was evaluated by Oil Red O staining. Transmission electron microscopy was employed to examine liver cell ultrastructure, and TUNEL staining was used to assess hepatocellular apoptosis. Transcriptome sequencing was conducted on liver tissues to identify key genes and pathways, which were further validated by Western blotting and immunohistochemistry.

**Results:**

Initial blood sampling revealed decreased serum zinc concentration in the PCOS group, alongside elevated levels of testosterone (T), luteinizing hormone (LH), alanine aminotransferase (ALT), aspartate aminotransferase (AST), triglycerides (TG), total cholesterol (TC), blood glucose, fasting insulin, and oral glucose tolerance test (OGTT) values. However, the levels of serum estrogen (E2) and follicle-stimulating hormone (FSH) in the PCOS group were significantly decreased. Markers of oxidative stress, including malondialdehyde (MDA), superoxide dismutase (SOD), glutathione disulfide/glutathione ratio (GSSG/GSH), glutathione peroxidase (GSH-PX), and catalase (CAT), were also increased. Zinc sulfate treatment effectively improved all these parameters. HE and Oil Red O staining confirmed that zinc sulfate mitigated high-fat diet and letrozole-induced fatty liver. Furthermore, zinc sulfate alleviated severe hepatocellular apoptosis and mitochondrial damage observed in PCOS rats. Transcriptomic analysis indicated that zinc sulfate primarily mitigated PCOS-related liver damage via the cholesterol synthesis pathway, and experimental validation demonstrated that zinc sulfate inhibited oxidative stress and apoptosis in liver cells through the NF-κB pathway.

**Conclusion:**

Our study demonstrates that zinc sulfate ameliorates liver oxidative stress and apoptosis in PCOS by modulating the NF-κB pathway, offering a novel therapeutic approach for managing PCOS-associated liver diseases.

## Introduction

Polycystic ovary syndrome (PCOS) is a prevalent endocrine disorder characterized by chronic anovulation and hyperandrogenemia (1), with an estimated prevalence of 11% to 13% (2). PCOS is a complex, multifactorial condition that encompasses metabolic, genetic, epigenetic, and environmental factors (3). Hyperandrogenemia (HA) and hyperinsulinemia (HI) are its primary pathological features (4). Insulin resistance (IR), defined as impaired insulin action in major target tissues such as muscle, adipose tissue, and liver, affects approximately 30% to 35% of PCOS patients, particularly those who are obese (5). Women diagnosed with PCOS, especially those exhibiting the HA phenotype, have a higher incidence of IR, which is exacerbated by obesity (6). Excessive androgens can induce IR, while the compensatory increase in insulin leading to HI promotes the accumulation of abdominal and visceral fat, further stimulating androgen secretion from the ovaries and adrenal glands in PCOS patients (7). The multifactorial nature of IR impairs glucose uptake and utilization by organs, tissues, and cells, ultimately perpetuating IR. The interplay between HI and HA resulting from IR creates a vicious cycle that exacerbates the pathophysiology of PCOS.

Metabolic dysfunction-associated steatotic liver disease (MASLD) redefines non-alcoholic fatty liver disease (NAFLD) and introduces a new nomenclature. The term metabolic dysfunction-associated steatohepatitis (MASH) has been proposed to replace non-alcoholic steatohepatitis (NASH). Studies have shown that 98% of NAFLD patients meet the MASLD criteria (8). PCOS is significantly associated with MASLD. Insulin plays a crucial role in regulating glucose and lipid metabolism, both directly and indirectly within the liver. Specifically, insulin promotes protein synthesis, glucose storage, and glycolysis while initiating lipid synthesis and storage. Conversely, it inhibits hepatic ketogenesis and gluconeogenesis. Women with PCOS have a fourfold higher risk of developing NAFLD compared to those without PCOS (9). Recent findings indicate that the pathophysiology of MASLD primarily involves lipotoxicity, hepatic immune dysregulation, hepatic and systemic insulin resistance, and gut microbiota imbalance, characterizing MASLD as a systemic metabolic disorder (10). Consequently, PCOS can be considered a disease related to systemic metabolic disorders. Research has demonstrated that hyperinsulinemia upregulates transcription factors involved in hepatic fat synthesis, such as SREBP-1c and ChREBP, thereby stimulating hepatic fat accumulation and predisposing women with PCOS to MASLD (11). Additionally, dysregulation of liver factors (primarily fetuin-A), fibroblast growth factor-21 (FGF-21), and selenoprotein P1 (SEPP1) contributes to the development of MASLD, alterations in lipid metabolism, and increased oxidative stress in women with PCOS. Given the strong association between insulin resistance and PCOS, emerging evidence suggests that liver tissue damage may play a significant role in the pathogenesis of PCOS (12). Preliminary experimental observations indicate that abnormal lipolysis and elevated circulating free fatty acid (FFA) levels promote lipid overload and hepatic accumulation, leading to lipotoxicity and the release of inflammatory cytokines, thereby exacerbating hepatic steatosis and insulin resistance (13). Furthermore, studies have found that a 7% reduction in body weight can decrease fat accumulation, alleviate inflammation, and improve liver histological damage (14).

Oxidative stress signifies an imbalance between the production of reactive oxygen species (ROS) and the body’s antioxidant defense mechanisms (15), leading to elevated ROS levels that damage mitochondrial membranes and compromise the effectiveness of the antioxidant system (16). PCOS pathogenesis is closely associated with oxidative stress, which primarily affects ovarian function, thereby diminishing oocyte quality and inducing granulosa cell apoptosis. This results in anovulation and luteal degeneration (17). Additionally, oxidative stress plays a crucial role in the progression from steatosis to NASH. Lipid accumulation in hepatocytes induces lipotoxicity by impairing oxidative phosphorylation and altering mitochondrial calcium influx, which leads to dysfunction of the electron transport chain and increased ROS generation. Lipotoxicity and mitochondrial dysfunction can trigger oxidative stress, endoplasmic reticulum (ER) stress, and elevated ROS levels in the liver, activating Kupffer cells to release inflammatory cytokines (8). Mitochondria serve as key regulators maintaining the balance between fatty acid β-oxidation, the electron transport chain, ATP production, and ROS (18). Abnormal mitochondrial activity disrupts this balance, resulting in reduced fatty acid metabolism and subsequent ROS production (16). Studies have shown that serum zinc ion levels are lower in PCOS patients (19). Zinc sulfate, acting as an antioxidant, can mitigate PCOS progression by modulating major cellular signaling pathways such as peroxisome proliferator-activated receptor alpha (PPAR-α) and lipid metabolism (20), insulin-like growth factor-1 (IGF-1), and insulin sensitivity (21). Consequently, we hypothesize that zinc sulfate may further ameliorate liver tissue damage by regulating insulin resistance and oxidative stress.

In this study, we investigated the potential of zinc sulfate to alleviate NAFLD in PCOS by modulating lipid metabolism and elucidating the underlying molecular mechanisms. Letrozole, an aromatase inhibitor, has been shown to increase testosterone levels and elevate hepatic triglyceride concentrations after 28 days of administration. Consequently, we utilized a combination of 1 mg/kg letrozole and a high-fat diet (HFD) to establish a PCOS rat model (22). The primary objective of this study was to examine the therapeutic effects of zinc sulfate on hepatic conditions in PCOS rats and to uncover the molecular pathways involved in treating PCOS-associated liver diseases.

## Materials and methods

### Ethics authorization

This study received approval from the Medical Ethics Committee of Shanxi Provincial People’s Hospital. The ethics approval number is No. 852 of the Provincial Medical Department.

### Experimental animals, drugs, and reagents

A total of 30 female Sprague-Dawley (SD) rats, aged 3 weeks and of SPF grade, were procured from the Animal Experiment Center of Shanxi Provincial People’s Hospital. The animals were maintained in a controlled environment with a temperature range of 23-25°C, relative humidity of 55%, and a 12-hour light-dark cycle, with ad libitum access to water. Following a one-week acclimatization period, the rats were randomly allocated into two groups: a control group (n=10) receiving 1% carboxymethyl cellulose (CMC) at 1 mg/kg, and a model group (n=20) receiving letrozole dissolved in 1% CMC at 1 mg/kg. The control group was fed a standard diet, while the model group was provided with a high-fat diet (D12492, Research Diets; 60.2% fat, 20.12% protein, total fat content 4.3%) ad libitum. Body weight was recorded weekly. Vaginal smears were examined starting from day 10 post-modeling. Disruption of the estrous cycle confirmed the establishment of the PCOS model. Subsequently, the model group was further divided into a model subgroup and a treatment subgroup. Both subgroups continued on the high-fat diet and letrozole regimen, while the treatment subgroup received zinc sulfate solution (1 ml per 200 g body weight) containing Zn2+ at a concentration of 15 mg/kg/day via oral gavage for four weeks. Experimental measurement of Zinc sulfate heptahydrate is based on previous literature reports (23, 24) to ensure animal safety and ethical principles.

Letrozole tablets (2.5 mg/tablet) were obtained from Yimeshu Company (Approval No.: National Drug Approval H20133109). Zinc sulfate heptahydrate was sourced from Solarbio (Catalog No. Z8090).

### Vaginal smears

On the 10th day of modeling and the 10th day of zinc sulfate administration, vaginal exfoliated cells from rats were collected. Swabs were gathered at a consistent time each day and subjected to HE staining for observation of the estrous cycle. The typical estrous cycle of mice spans 4–5 days, encompassing proestrus, estrus, metestrus, and diestrus phases. Three types of cells were identified. Cells with a round shape possessing nuclei were classified as epithelial cells, cells with an irregular shape lacking nuclei were termed keratinized cells, and small round cells were identified as white blood cells. The stages of the estrus cycle were determined based on the ratio among these cells. In the proestrus stage, the smear is primarily composed of nucleated epithelial cells; during the estrus stage, keratinized epithelial cells predominate; in the metestrus stage, the smear predominantly consists of a mixture of epithelial cells and leukocytes; in the anestrus stage, leukocytes are the majority, while nucleated epithelial cells are present in lesser amounts. Disruption of the estrous cycle is characterized by deviations from the normal cyclical changes.

### Specimen collection

Following the establishment of the model, rats were subjected to an overnight fast. Blood was collected from the tail vein to measure the OGTT levels. On the subsequent morning, rats were anesthetized using isoflurane, and approximately 3–4 mL of blood was withdrawn from the abdominal aorta into EDTA-coated tubes. Following anesthesia, the rats were euthanized. Blood samples were centrifuged at 3000 rpm for 15 minutes at 4°C, and the resulting serum was stored at -80°C for biochemical analysis. Ovarian and liver tissues were excised. A portion of these tissues was fixed in 4% paraformaldehyde for 24 hours, while the remaining liver tissue was stored at -80°C for further experimentation.

### Biochemical analysis

The concentrations of testosterone (T), zinc ions, alanine aminotransferase (ALT), aspartate aminotransferase (AST), fasting blood glucose (FBG), fasting insulin (FINS), triglycerides (TG), malondialdehyde (MDA), superoxide dismutase (SOD), glutathione disulfide to glutathione ratio (GSSG/GSH), glutathione peroxidase (GSH-PX), and catalase (CAT) were measured using enzyme-linked immunosorbent assay (ELISA) kits (Shanghai Biotechnology Co., Ltd., Shanghai, China) according to the manufacturer’s instructions. The homeostasis model assessment of insulin resistance (HOMA-IR) was calculated using the formula: FBG (mmol/L) × FINS (mU/L)/22.5.

### Oil red O staining and hematoxylin-eosin staining

Following fixation with 4% paraformaldehyde, the dehydrated liver tissues were sequentially immersed twice in a sucrose solution of increasing concentration (15-30%) at 4°C. Subsequently, the tissue samples were embedded in an embedding agent and sectioned into 8-micrometer slices using an automated microtome. The sections were then stained with Oil Red O, followed by counterstaining with hematoxylin. After three washes with distilled water, the sections were mounted with glycerol gelatin and examined under a microscope for lipid droplet visualization. Additionally, ovaries and selected liver tissues were fixed in paraformaldehyde and processed for paraffin embedding. Sections of both tissues (5 μm) were stained with hematoxylin and eosin (HE) following the manufacturer’s protocol (Wuhan Service Biotechnology Co., Ltd., Wuhan, China) and examined microscopically to evaluate tissue and cellular structures.

### Detection of apoptosis by TUNEL assay

Deparaffinize the sections to water following this protocol: immerse in deparaffinizing solution I for 30 minutes, then in deparaffinizing solution II for another 30 minutes. Subsequently, rinse in absolute ethanol twice for 5 minutes each, followed by 5-minute immersions in 95%, 85%, and 75% alcohol solutions respectively. Finally, rinse with running tap water for 5 minutes. Conduct microwave antigen retrieval using citrate buffer for 10 minutes, followed by three 5-minute washes with PBS. In a dark environment, prepare the fluorescent TUNEL incubation solution at a ratio of A:B = 1:30 and incubate at 37°C for 60 minutes. Wash with PBS, stain nuclei with DAPI for 15 minutes, rinse again with PBS, mount with glycerol, and store at -20°C. All reagents should be freshly prepared and used under light-protected conditions. For each section, more than three high-magnification fields were randomly selected. Normal cells and apoptotic cells in the pictures were counted separately by manual counting, and the percentage of apoptotic cells was calculated as apoptotic cells/(normal cells + apoptotic cells) ×100%.

### Transmission electron microscopy

Fixation: Extract 1 mm³ of liver tissue and perform initial fixation using 2.5% glutaraldehyde, followed by secondary fixation with 1% osmium tetroxide. Dehydration: Gradually dehydrate the sample using acetone in increasing concentrations (30%, 50%, 70%, 80%, 90%, 95%, and 100%), with three changes at 100% concentration. Impregnation: Sequentially impregnate the sample with a mixture of acetone and Epon-812 embedding agent in ratios of 3:1, 1:1, and 1:3. Embedding: Embed the sample in pure Epon-812 embedding agent. Semi-thin and ultra-thin sectioning: Examine semi-thin sections under a light microscope to identify the region of interest in the rat small intestinal mucosal epithelium. Subsequently, prepare 60–90 nm ultra-thin sections using an ultramicrotome and place them on copper grids. Staining: Stain the ultra-thin sections first with uranyl acetate for 10–15 minutes, followed by lead citrate for 1–2 minutes at room temperature. Finally, capture images using transmission electron microscopy (TEM).

### Transcriptome sequencing

Liver tissues were harvested from three rats in each group for analysis. Total RNA was extracted and purified using TRIzol (Thermo Fisher, cat. 15596018) according to the manufacturer’s instructions. The concentration and purity of total RNA were assessed using a NanoDrop ND-1000 spectrophotometer (NanoDrop Technologies, Wilmington, DE, USA), while RNA integrity was evaluated using an Agilent Bioanalyzer 2100 (Agilent Technologies, CA, USA). Samples meeting the criteria of RNA concentration > 50 ng/μL, RIN value > 7.0, and total RNA amount > 1 μg were selected for downstream applications. Polyadenylated mRNA was selectively captured from total RNA using oligo(dT)-coated magnetic beads (Dynabeads Oligo(dT), Thermo Fisher Scientific, cat. 25-61005) through two rounds of purification. The isolated mRNA was fragmented at high temperature using the NEBNext Magnesium RNA Fragmentation Module (New England Biolabs, cat. E6150S) for 5–7 minutes at 94°C. Subsequently, the fragmented RNA was reverse transcribed into cDNA using Invitrogen SuperScript II Reverse Transcriptase (cat. 1896649). Second-strand synthesis was performed using E. coli DNA polymerase I (NEB, cat. M0209) and RNase H (NEB, cat. M0297), incorporating dUTP (Thermo Fisher, cat. R0133) during this process to generate blunt ends on double-stranded DNA, followed by addition of an A base to each end to facilitate ligation with adapters containing a T base. The fragments were then size-selected and purified using magnetic beads. UDG enzyme (NEB, cat. M0280) was used to digest the second strand, followed by PCR amplification: pre-denaturation at 95°C for 3 minutes, denaturation at 98°C for 15 seconds per cycle over 8 cycles, annealing at 60°C for 15 seconds, extension at 72°C for 30 seconds, and final extension at 72°C for 5 minutes, resulting in a strand-specific library with a fragment size of 300 bp ± 50 bp. Finally, the library was sequenced bidirectionally on an Illumina Novaseq 6000 platform using the PE150 mode. Raw sequencing data underwent quality control, and gene (transcript) differential expression analysis was based on read counts. P-values were calculated using a negative binomial distribution model. For samples with biological replicates, DESeq2 was employed for analysis; for those without replicates or for multiple comparisons, edgeR was utilized. The p-values were adjusted using the Benjamini-Hochberg method to obtain q-values (FDR values, p.adj values). Fold change was determined as the ratio of average expression levels between the experimental and control groups. Gene Ontology (GO) and Kyoto Encyclopedia of Genes and Genomes (KEGG) analyses were conducted to interpret the RNA-seq results.

### Molecular docking

AlphaFold3 is capable of predicting complexes involving proteins, nucleic acids, small molecules, ions, and modified protein residues, as well as antibody-antigen interactions. To predict the interaction between small molecules and the protein encoded by the TOP gene, first retrieve the FASTA sequence of this protein from the NCBI website. Then, utilize AlphaFold3 (available at https://alphafoldserver.com/) for the prediction, and employ PyMOL to visualize and refine the docking results.

### Western blotting

Approximately 50 mg of liver tissue was harvested from each rat on ice. Total protein extraction from the ovarian tissue was performed using RIPA lysis buffer supplemented with protease and phosphatase inhibitors (1 ml lysis buffer per 50 mg tissue). Protein concentration was quantified using the BCA assay. Proteins were subsequently separated by 10% SDS-PAGE and electrotransferred onto PVDF membranes. The membranes were blocked with 5% non-fat milk at room temperature for 2 hours, followed by washing. They were then incubated overnight at 4°C with the primary antibody. On the following day, after additional washing, the membranes were incubated with a species-matched secondary antibody for 1 hour at room temperature, followed by another wash. Blots were visualized using a high-sensitivity chemiluminescent substrate and imaged with the SCG-W3000 chemiluminescence system (Servicebio, China). Band intensities were quantified using ImageJ software.

### Immunohistochemical staining

Systematic pretreatment procedures were conducted on paraffin-embedded rat liver sections, encompassing dewaxing, dehydration, antigen retrieval, blocking of endogenous peroxidase activity, and incubation with primary antibodies. In the immunohistochemistry (IHC) experiments, the primary antibodies utilized were phospho-NFκB and phospho-IκB. Subsequently, an enzyme-labeled goat anti-mouse/rabbit IgG polymer was employed as the secondary antibody. The sections were then subjected to gradient alcohol dehydration and xylene clarification. Finally, the sections were sealed using neutral gum.

### Statistical analysis

The results are presented as the mean ± S.D. for continuous variables. Statistical data were determined by t test and one-way ANOVA using Graphpad Prism 9.5 software. A pvalue <0.05 was considered statistically significant. All experiments were replicated three times. (* means that P<0.05,** means that P<0.01, *** means that P<0.001, **** means that P<0.0001).

## Results

### Rat models and sex hormone levels

**Figure 1:**
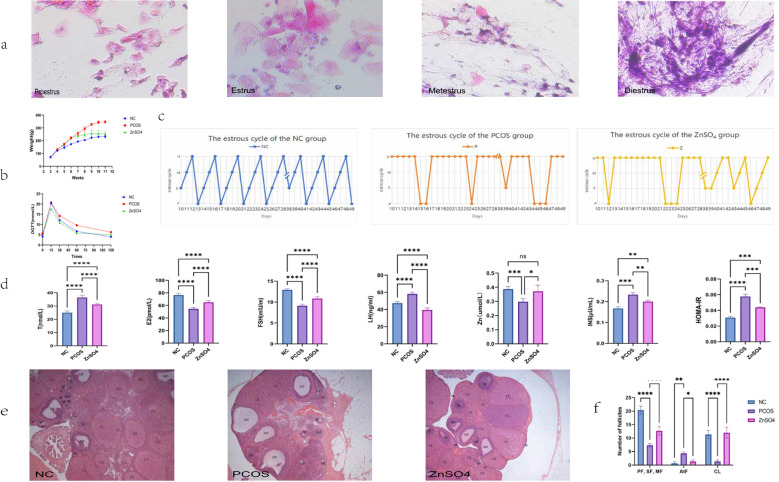
Effects of Zinc Sulfate on Body Weight, Ovarian Pathology, and Biochemical Parameters in Polycystic Ovary Syndrome Rats. **(a)** Vaginal Smear Staining. **(b)** Changes in Body Weight and OGTT Results. **(c)** The changes in the emotional cycle (P, proestrus; E, estrus; M, metestrus; D, diestrus). **(d)** Serum Levels of Testosterone, luteinizing hormone, estrogen, follicle-stimulating hormone, Zinc Ions, Fasting Insulin, and HOMA-IR. Data are expressed as mean ± standard (n=10). **(e)** Histopathological Observation of Ovarian Tissue via HE Staining and Ovarian morphology score. **(f)** Number of follicles (PF, primary follicle; SF, secondary follicle; MF, mature follicle; AtF, atretic follicle; CL,corpus luteum). Data are expressed as mean ± standard (n=3). * means that P<0.05,** means that P<0.01, *** means that P<0.001, **** means that P<0.0001.

On the 10th day of administration of HFD and letrozole, vaginal smears were collected from SD rats to examine vaginal exfoliated cells. Rats in the control group exhibited regular estrous cycles (**Figure 1a**). In contrast, rats in the model group displayed endocrine and estrous cycle disorders (**Figures 1b, c**), with vaginal smears indicating that all cells were in the diestrus or interestrus phase, suggesting successful establishment of the PCOS rat model. Following 21 days of zinc sulfate treatment, the estrous cycles of rats in the treatment group gradually normalized. ELISA analysis revealed significant improvements in testosterone, luteinizing hormone, estrogen, follicle-stimulating hormone, zinc ion concentrations, fasting insulin levels, and OGTT results in the treatment group (**Figure 1d**). Additionally, weight gain was higher in the model group compared to the control group but was significantly reduced following zinc sulfate treatment. According to the results of ovarian morphological analysis, it was found that the follicles at all levels in the ovaries of rats in the NC group grew and developed well, the granulosa cells were neatly and closely arranged, and the number of corpus luteum was relatively large. Compared with the NC group, there were more cystic dilated-like follicles in the ovaries of the P group. The granulosa cells were sparsely arranged, the number of layers decreased, oocytes were absent, and the number of lutees and the number of follicles at all levels were significantly lower. Compared with NC, the number of cystic dilatable follicles in the ovaries of rats in group Z was significantly reduced. Follicles at different developmental stages could be observed. The ovarian structure partially recovered. The granulosa cell layer in the dominant follicles was thicker and denser, and the local morphology was similar to that of the control group (**Figures 1e, f**).

### Serum indexes of rats

**Figure 2:**
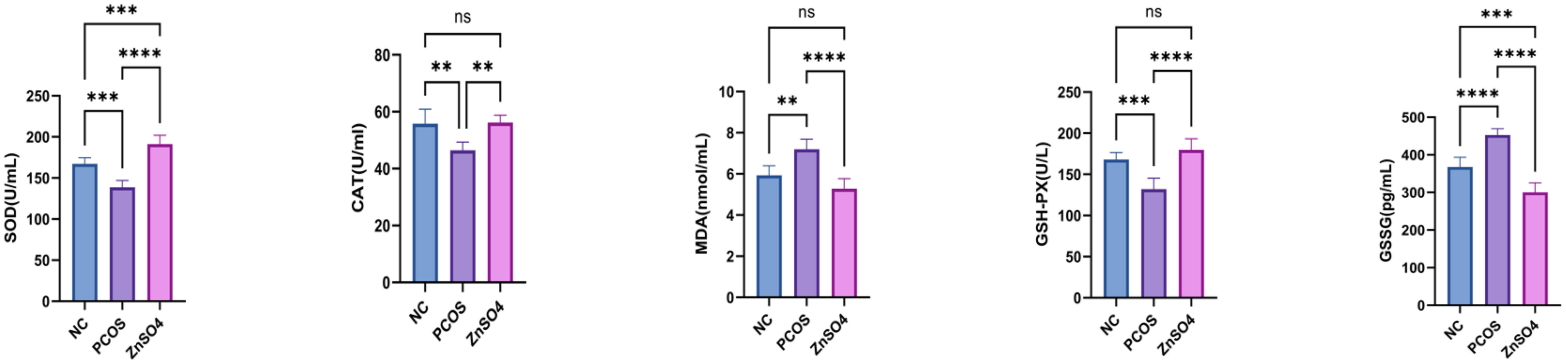
Effects of Zinc Sulfate on Serum Oxidative Stress Indicators in PCOS Rats. Zinc Sulfate Attenuates Oxidative Stress in PCOS Rats (SOD activity, CAT activity, Serum MDA levels, GSH-Px activity and GSSG/GSH ratio). Data are expressed as mean ± standard (n=10). ns means that P > 0.05, means that P<0.01, *** means that P<0.001, **** means that P<0.0001.

To investigate the changes in OS in PCOS rats, we used the biochemical process to assess the concentration of the lipid peroxidation product MDA, the GSSG ratio and antioxidant enzymes SOD, GSH-PX and CAT in serum samples. Significantly elevated levels of MDA, the GSS ratio and decreased levels of SOD, GSH-PX and CAT were found in PCOS patient (**Figure 2**). These results jointly indicate that PCOS is associated with intensified lipid peroxidation (elevated MDA) and the collapse of antioxidant defense (decreased SOD, GSH- PX, and CAT), confirming the key role of OS in the pathogenesis of PCOS.

### Zinc sulfate improves hepatic steatosis and liver function in SD rats

**Figure 3:**
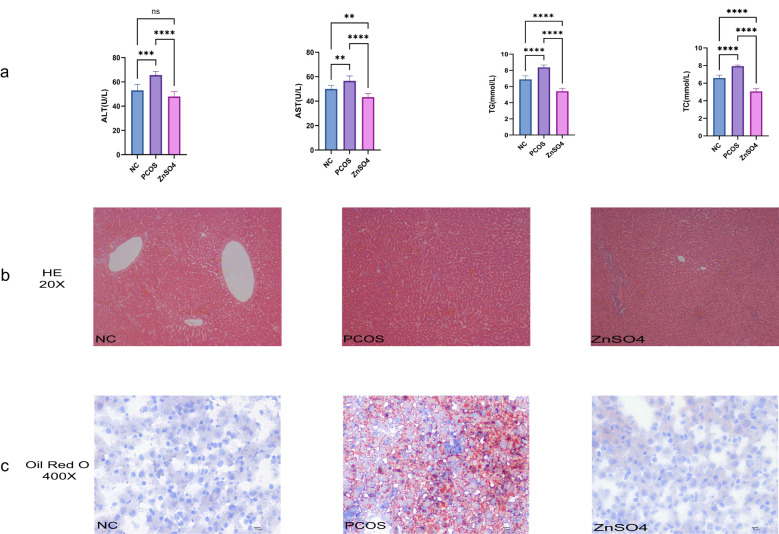
Impact of Zinc Sulfate on Liver Function in PCOS Rats. **(a)** Alterations in ALT, AST, TG, and TC Levels. Data are expressed as mean ± standard (n=10). ns means that P > 0.05, ** means that P<0.01, *** means that P<0.001, **** means that P<0.0001. **(b)** Histopathological Examination of Liver Tissue via HE Staining. **(c)** Detection of Lipid Droplets in Liver Tissue Using Oil Red O Staining.

The levels of ALT, AST, triglycerides, and total cholesterol in the model group rats were significantly elevated compared to those in the control group (**Figure 3a**). However, zinc sulfate treatment reversed this trend. Additionally, both HE staining (**Figure 3b**) and Oil Red O staining (**Figure 3c**) revealed pronounced lipid accumulation and disordered hepatocyte structure in the livers of the model group rats. These findings suggest a potential association between increased OS levels and liver function abnormalities in PCOS. Collectively, these results indicate that zinc sulfate exhibits a significant protective effect against hepatic steatosis induced by HFD and letrozole.

### Zinc sulfate improves apoptosis in liver tissue

**Figure 4:**
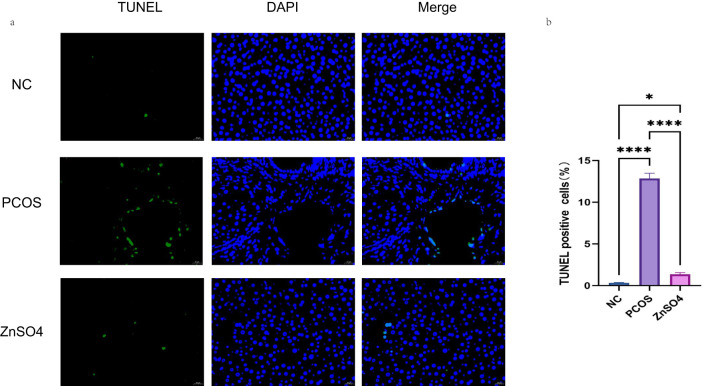
Tunel staining was used to observe the effect of zinc sulfate on the apoptosis of liver cells. **(a)** Fluorescence staining (Green represents apoptotic cells and blue represents the cell nucleus). **(b)** The percentage of apoptotic cells. Data are expressed as mean ± standard (n=3). ns means that P > 0.05, * means that P<0.05, **** means that P<0.0001.

The TUNEL staining results demonstrated that apoptosis was evident in the liver cells of the PCOS group (**Figure 4a**). However, zinc sulfate treatment markedly ameliorated this apoptosis. Specifically, the apoptosis rates for the model group and the treatment group were 12.84% and 0.83% (**Figure 4b**), respectively. Compared with the control group, the model group exhibited a significant increase in apoptosis rate, whereas the treatment group showed a relative improvement.

### Zinc sulfate improves NAFLD in PCOS by enhancing mitochondrial function

**Figure 5:**
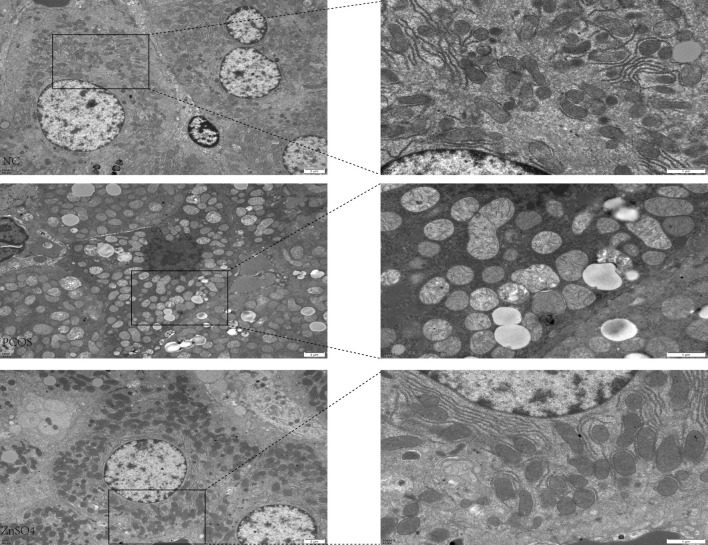
Transmission electron microscopy observation of the effect of zinc sulfate on liver cells.

In the control group, all hepatocytes exhibited normal nuclei characterized by an oval shape and intact, continuous nuclear membranes. Most mitochondria were either round or rod-shaped, with distinct cristae visible in proximity to the nuclei. In contrast, in the PCOS rat model, hepatocyte apoptosis was evident, marked by darker and irregularly serrated nuclei, significantly swollen mitochondria with reduced cristae, and the presence of lipid droplet vacuoles. These pathological changes were notably ameliorated in the treatment group (**Figure 5**).

### Transcriptome sequencing of the liver

**Figure 6:**
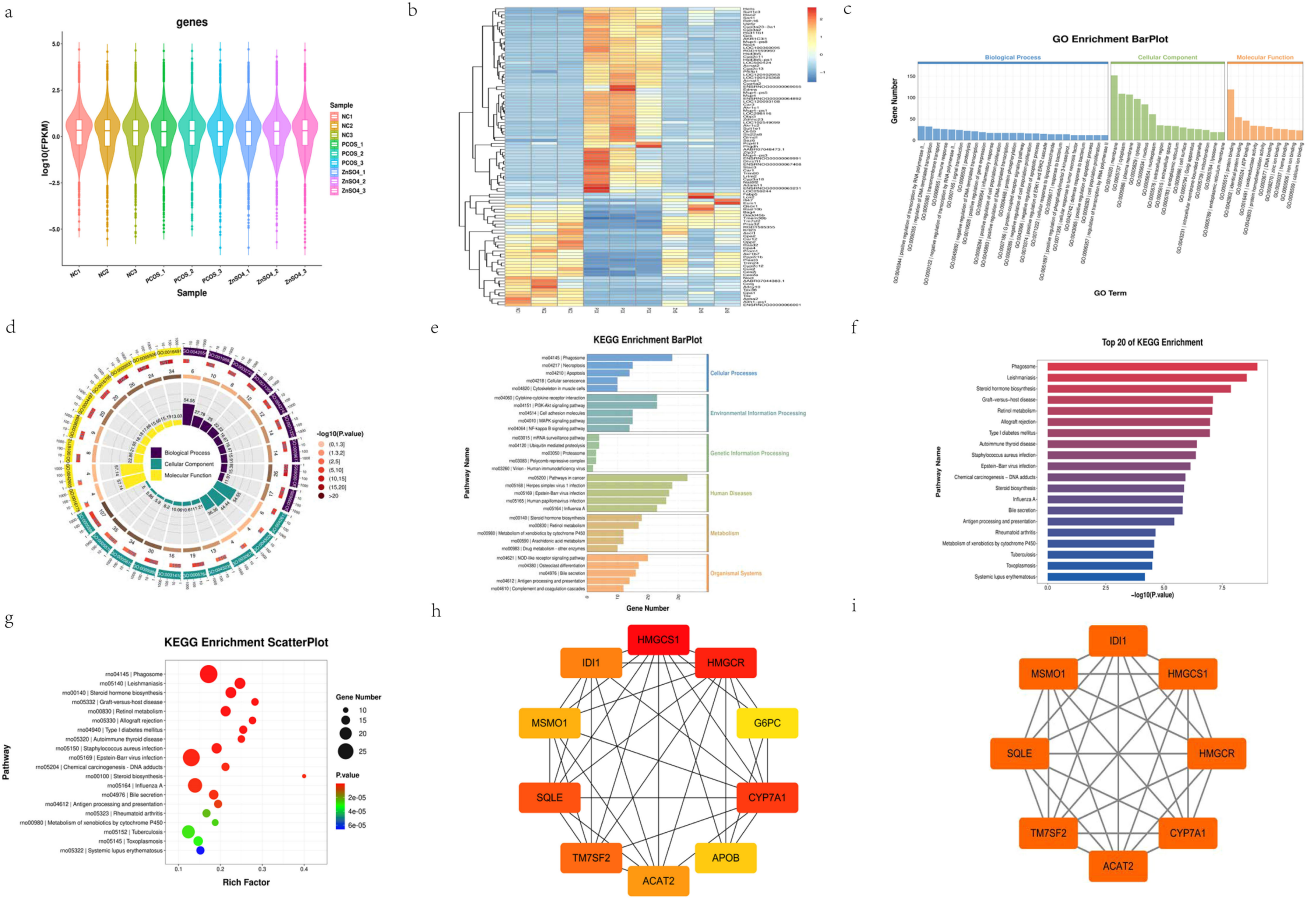
Transcriptional Profiling of Liver Tissues. **(a)** Statistical distribution of gene expression levels across samples. **(b)** Heatmap illustrating differentially expressed genes. **(c)** GO enrichment analysis of differentially expressed genes. **(d)** String network diagram derived from GO enrichment analysis. **(e)** Bar chart summarizing KEGG pathway enrichment analysis for differentially expressed genes. **(f)** Bar chart highlighting KEGG pathway enrichment analysis of the top 20 genes. **(g)** Dot plot depicting the top 20 signaling pathways identified through KEGG enrichment analysis. **(h)** Top 10 differentially expressed genes as determined by cytoHubba module analysis. **(i)** Genes within cluster 1 identified via MCODE module analysis.

Through transcriptome sequencing of three groups (**Figure 6a**), a total of 488 differentially expressed genes (DEGs) were identified (**Figure 6b**). Gene Ontology (GO) and Kyoto Encyclopedia of Genes and Genomes (KEGG) enrichment analyses revealed that at the cellular component (CC) level, these genes were primarily enriched in membrane, cytoplasm, and plasma membrane categories. At the molecular function (MF) level, they were predominantly enriched in protein binding, ATP binding, and oxidoreductase activity. At the biological process (BP) level, the DEGs were significantly enriched in processes such as positive regulation of transcription elongation by RNA polymerase II, regulation of DNA-templated transcription, and transmembrane transport (**Figures 6c, d**). KEGG pathway analysis indicated that the DEGs were mainly enriched in apoptosis-related pathways and oxidative stress pathways, which are consistent with the pathogenesis of PCOS (**Figures 6e–g**). Furthermore, protein-protein interaction (PPI) network analysis was conducted on the DEGs, and the results were imported into Cytoscape 3.7.2 for cytoHubba and MCODE module analysis (**Figure 6h**). The intersection of the top-ranking genes from both modules yielded eight significant genes: CYP7A1, HMGCS1, ACAT2, MSMO1, SQLE, TM7SF2, HMGCR, and IDI1 (**Figure 6i**). These genes are all significantly associated with steroid hormone synthesis.

### Molecular docking

**Figure 7:**
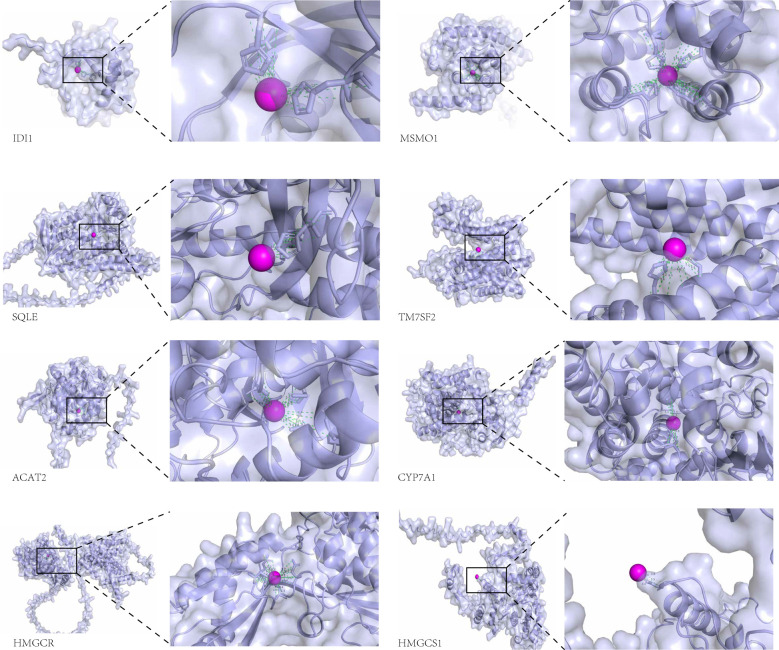
Visual representation of the docking analysis of the differentially expressed genes CYP7A1, HMGCS1, ACAT2, MSMO1, SQLE, TM7SF2, HMGCR, and IDI1 with Zn^2+^.

The molecular docking analysis in this study was intended to provide a hypothesized direction for subsequent mechanism studies, rather than as functional evidence of direct binding of zinc to target proteins. The interaction between the protein molecules of these eight genes and zinc ions was examined, revealing a significant association. The figure illustrates the notable interaction between each protein and zinc ions (**Figure 7**).

### Zinc sulfate alleviated hepatic injury by activating the NF-κB pathways in PCOS

**Figure 8:**
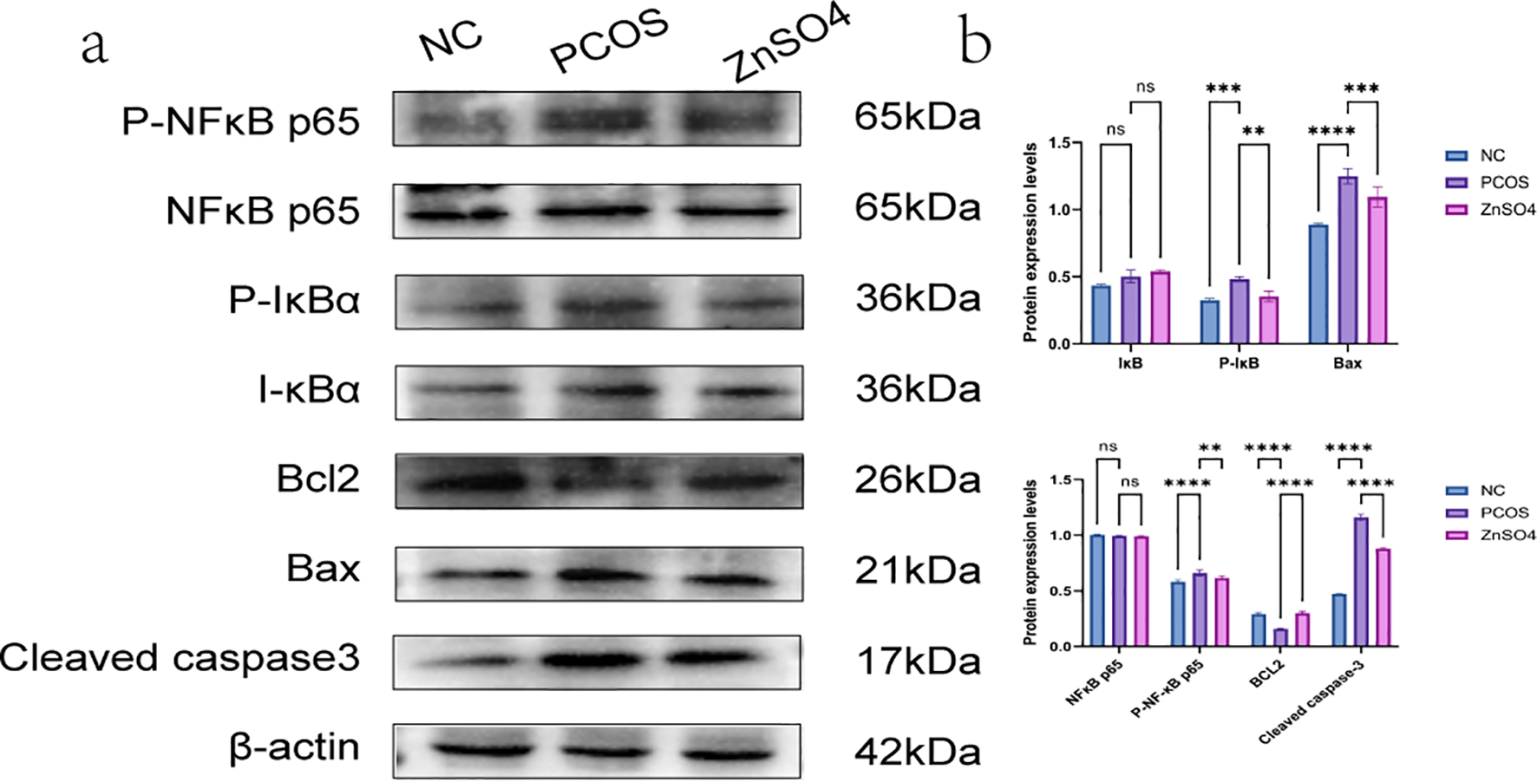
Expression of P-NFκB p65, P-IκB, NFκB p65, IκB, BCL2, Bax and Cleaved caspase3 in liver tissue by Western blotting. **(a)** Representative images of western blot. **(b)** Quantitative analysis of protein expression. On the same membrane, each internal reference is used for homogenization treatment. Data are expressed as mean ± standard (n=3). ns means that P > 0.05, ** means that P<0.01, *** means that P<0.001, **** means that P<0.0001.

**Figure 9:**
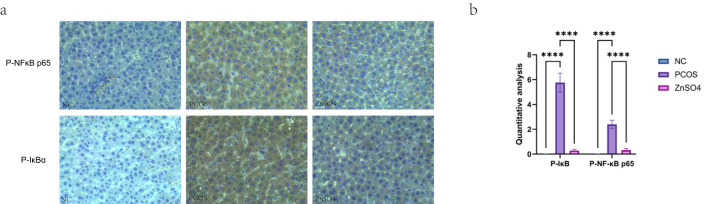
Expression of P-NFκB p65 and P-IκB in liver tissues by immunohistochemistry. **(a)**. Representative images of immunohistochemical staining. **(b)** Quantitative analysis of protein expression. On the same membrane, each internal reference is used for homogenization treatment. Data are expressed as mean ± standard (n=3). ns means that P > 0.05, **** means that P<0.0001.

Western blot analysis demonstrated that the phosphorylation levels of NF-κB p65 and IκB in liver tissues from rats in the PCOS group were significantly elevated, whereas the expression levels of non-phosphorylated NF-κB p65 and IκB remained unchanged. Additionally, the expression of the anti-apoptotic protein BCL2 was reduced, while the pro-apoptotic proteins Bax and Cleaved caspase-3 showed increased expression. These findings suggest that the treatment can modulate the phosphorylation status of the NF-κB pathway, thereby mitigating oxidative stress and apoptosis in hepatic cells (**Figure 8**). Immunohistochemical staining further confirmed the upregulation of P-NFκB p65 and P-IκB in the model group, indicating that PCOS activates the NF-κB signaling pathway, contributing to cellular oxidative stress (**Figure 9**).

## Discussion

PCOS is a prevalent endocrine disorder characterized by hyperandrogenemia, polycystic ovaries, menstrual irregularities, and infertility (25). The rat model induced by letrozole in conjunction with a HFD exhibited estrous cycle disturbances, hyperandrogenemia, polycystic ovarian morphology, and impaired glucose and lipid metabolism, confirming the successful establishment of the PCOS rat model. A cross-sectional ultrasound study conducted in the Chinese population revealed that the prevalence of NAFLD among PCOS patients was 56%, compared to 38% in healthy women (26, 27). These findings indicate a significant association between PCOS and an increased prevalence of NAFLD. To date, no definitive pharmacological interventions have been identified to prevent the development of fatty liver disease in PCOS patients.

Sarkar et al. (28) conducted a detailed analysis of liver biopsies from women diagnosed with PCOS and observed that their livers exhibited more pronounced edema and NASH compared to those of women without PCOS. Furthermore, patients with HA demonstrated even more severe hepatic manifestations. Zhang et al. (29) reported that hCG-induced hyperandrogenic rats exhibited significantly exacerbated liver inflammation, and the NAFLD observed in PCOS patients was strongly associated with hyperandrogenemia and insulin resistance.

Consistent findings were observed in our study. Compared to the control group, the serum levels of ALT, AST, TC, and TG in PCOS rats were markedly elevated. Histological examination via HE staining and Oil Red O staining revealed that liver tissues from PCOS rats exhibited hepatocellular swelling and spheroidization, along with inflammatory cell infiltration and an increase in lipid droplets. Additionally, all oxidative stress markers in PCOS rats were significantly altered, indicating the critical role of oxidative stress in the pathogenesis and progression of PCOS.

Androgens may exacerbate TG accumulation by modulating TG secretion in hepatocytes, thereby aggravating hepatic steatosis. Excessive accumulation of TG will intensify the production of ROS, resulting in both compensatory and uncompensatory oxidative stress, activation of inflammatory response, and aggravation of liver cell damage (30, 31). The increase of antioxidant enzyme activity may not simply reflect the severity of oxidative damage, but may represent the compensatory protective response triggered by the body in response to oxidative stress. Although there may be a compensatory increase in antioxidant enzyme activity, a significant increase in markers of oxidative damage was observed in this study, suggesting that the compensatory capacity of the antioxidant system is insufficient to completely offset ROS overproduction. This imbalance may be due to the persistence of chronic inflammation and lipid toxic microenvironment in the PCOS state, leading to long-term overload of the antioxidant defense system, and eventually leading to pathological outcomes dominated by oxidative damage. Concurrently, apoptosis and oxidative stress-induced damage to hepatocytes impair their ability to process blood glucose, resulting in insulin resistance and further promoting the progression of PCOS. Elevated fasting insulin levels increase the risk of developing PCOS. Obesity upregulates the expression of pro-inflammatory genes, subsequently increasing the production of pro-inflammatory cytokines in the liver and inducing systemic insulin resistance (32, 33). A study involving 3,398 participants from the National Health and Nutrition Examination Survey (NHANES) conducted between 2011 and 2016 demonstrated a positive linear correlation between serum zinc concentration and the likelihood of developing NAFLD (34). Research has indicated that supplementing with 30 mg of zinc daily for 8 weeks may elevate serum zinc levels in NAFLD patients while reducing AST, TC, and LDL-C levels, suggesting the need for further investigation (35). Zinc sulfate, used as an antioxidant to mitigate oxidative stress, was found to significantly improve lipid deposition and liver function in rats with PCOS.

The selected top genes (CYP7A1, HMGCS1, ACAT2, MSMO1, SQLE, TM7SF2, HMGCR, IDI1) are predominantly key rate-limiting enzymes in the cholesterol biosynthesis pathway and are implicated in various physiological and pathological processes. Specifically, HMGCS1 catalyzes the conversion of acetyl-CoA to 3-hydroxy-3-methylglutaryl-CoA, marking the initial step of cholesterol synthesis (36). HMGCS1 is up-regulated by NRF2 (37), which is a marker of oxidative stress. We speculate that zinc ions may affect HMGCS1 in the NFF2 pathway, thereby regulating the occurrence of oxidative stress and reducing the damage of hepatocytes. HMGCR facilitates the conversion of HMG-CoA to mevalonate, which is a critical intermediate in the final stages of cholesterol synthesis. Dysfunctional HMGCR has been associated with lipid metabolism disorders observed in patients with PCOS (38). Studies have shown that estrogen can bind to the ERE region of HMGCR promoter to activate HMGCR expression and induce the increase of human serum total cholesterol and LDL (39), while after liver steatosis and inflammation, its ability to inactivate estrogen is reduced, and positive feedback activates HMGCR expression and promotes lipid deposition in the bile.CYP7A1 plays a pivotal role in converting cholesterol into bile acids, serving as one of the liver’s primary mechanisms for cholesterol clearance (40). Zinc ions can accelerate reverse cholesterol transport and enhance cholesterol elimination by antagonizing the CYP7A1-activated FXR pathway (41). TM7SF2, also known as Δ14-sterol reductase (C14SR), functions as a sterol reductase that reduces specific carbon-carbon double bonds in the sterol moiety during sterol biosynthesis. This gene is crucial in cell proliferation, apoptosis, metabolic regulation, and disease development (42). An animal experiment (43)] found that Tm7sf2 gene can promote adipocyte differentiation and improve insulin sensitivity in mouse embryonic fibroblasts. However, zinc ions can treat hepatic insulin resistance in PCOS, which was demonstrated in this study. Therefore, we hypothesized that zinc ions affect TM7SF2 gene expression, possibly through improving insulin sensitivity. MSMO1, a key factor in mitochondrial translation, may significantly contribute to the pathogenesis of PCOS (44). Estrogen can directly promote the transcription of MSMO1 through estrogen receptorα (45). The possible mechanism is similar to that of HMGCR, both of which stimulate gene expression by estrogen and then affect lipid deposition. SQLE, an essential ribosomal component, primarily focuses on protein synthesis and cell biology (46). Long-term intake of a diet high in plant protein improves serum and liver lipid and cholesterol accumulation by increasing the abundance of the trichospirochaeta family and serum levels of 12, 13-dihome and inhibiting SQLE expression (47). This study may also affect the lipid metabolism by influencing the intestinal flora and then regulating gene expression through some mechanism, which can be further studied in the future. IDI1, a cytoplasmic enzyme, participates in the biosynthesis of isoprenoids, including cholesterol (48). IDI1 gene expression was significantly increased in alveolar type II cells stimulated by LPS (49), suggesting that IDI1 may be primarily involved in inflammation and immune responses during cholesterol synthesis. ACAT2 is central to cholesterol metabolism. Overexpression of Acat2 inhibits the metabolic pathways of fatty acids, glucose and ketones, but promotes cholesterol metabolism and changes the bile acid pool and composition of liver (50) which is of great significance in the study of bile acid metabolic pathways. Cholesterol is a vital component of cell membranes. Elevated cholesterol synthesis can lead to lipid deposition, excessive fat accumulation, and subsequent lipotoxicity in the liver, resulting in impaired liver function and reduced lipid transport capacity.

Simultaneously, zinc sulfate exerts a significant effect on ameliorating liver cell apoptosis and mitochondrial oxidative stress injury, which aligns with our anticipated outcomes. Furthermore, zinc sulfate mitigates oxidative stress injury in PCOS rats via the NF-κB signaling pathways.

In the model group, we observed phosphorylation of NF-κB subunit p65 and IκBα in liver tissues, indicating activation of the classical NF-κB pathway and suggesting an elevated oxidative stress level. An increase in ROS can substitute for IκBα phosphorylation to activate NF-κB (51). The p50-p65 heterodimer plays a crucial role in the classical NF-κB pathway (52). Under normal conditions, these dimers reside in the cytoplasm bound to IκB (53). Activation of the classical NF-κB pathway involves the phosphorylation of IκBα, leading to the release of the p50-p65 dimer and subsequent phosphorylation of the p65 subunit. This process facilitates the translocation of the heterodimer to the nucleus, thereby activating the transcription of target genes. Phosphorylated p65 translocates from the cytoplasm to the nucleus, activating the NF-κB pathway (54), which results in histone and p65 acetylation and promotes the transcription of target genes (55). Liu et al. demonstrated that semaglutide can alleviate ovarian tissue inflammation and improve PCOS via the AMPK/SIRT1/NF-κB signaling pathway (56). Additionally, Jin et al. found that cuminin inhibits DHEA-induced phosphorylation of IκBα and NF-κB p65, thereby reducing the transcriptional activity of NF-κB and alleviating PCOS symptoms (57).

A meta-regression analysis revealed that the increasing prevalence of NAFLD is significantly associated with the rising prevalence of metabolic syndrome, as well as elevated levels of HOMA-IR, free androgen index, and total testosterone (58). These findings underscore the importance for clinicians to consider early screening for metabolic-related fatty liver disease and to implement preventive measures against its potential consequences. Our study confirmed that a HFD combined with letrozole can effectively establish pathological models of PCOS and NAFLD. Zinc sulfate mitigated hepatocyte apoptosis by alleviating mitochondrial oxidative stress injury in cells, thereby ameliorating non-alcoholic fatty liver disease in rats with PCOS, thus offering a novel therapeutic strategy for liver diseases associated with PCOS. However, this study was conducted solely at the animal level, and further validation at the cellular level is warranted. Multi-center prospective population studies are also needed to verify the availability of zinc sulfate. The inclusion of only 3 animals in the control group in the transcriptomic analysis may reduce the statistical power and increase the interference of individual differences in the results. Although we used rigorous bioinformatic screening to ensure the reliability of differential genes, small sample sizes may affect the detection sensitivity of some transcripts with low abundance or weak expression changes. Future studies need to expand the sample size to verify the generalizability of the current findings.

## Conclusion

In our study, we employed letrozole and a high-fat diet to establish a model of PCOS complicated with NAFLD. We investigated the potential of zinc sulfate to further ameliorate liver oxidative stress injury and reduce NAFLD via modulation of the NF-κB pathway. Our findings demonstrated that zinc sulfate not only improved sex hormone levels but also mitigated lipid accumulation and hepatocyte apoptosis in the liver, while enhancing mitochondrial oxidative function. This study suggests that zinc sulfate may represent a novel therapeutic strategy for preventing NAFLD in patients with PCOS.

## Data Availability

The datasets presented in this article are not readily available due to copyright restrictions. Requests to access the datasets should be directed to the corresponding author.

## Glossary

PCOS: polycystic ovary syndrome
NAFLD: non-alcoholic fatty liver disease
HE: hematoxylin and eosin
T: testosterone
E2: estradiol
FSH: follicle-stimulating hormone
LH: luteinizing hormone
ALT: alanine aminotransferase
AST: aspartate aminotransferase
TG: triglycerides
TC: total cholesterol
OGTT: oral glucose tolerance test
MDA: malondialdehyde
SOD: superoxide dismutase
GSSG/GSH: glutathione disulfide/glutathione ratio
GSH-PX: glutathione peroxidase
CAT: catalase
HA: hyperandrogenemia
HI: hyperinsulinemia
IR: insulin resistance
MASLD: metabolic dysfunction-associated steatotic liver disease
MASH: metabolic dysfunction-associated steatohepatitis
NASH: non-alcoholic steatohepatitis
FGF-21: fibroblast growth factor-21;SEPP1, selenoprotein P1
FFA: free fatty acid
ROS: reactive oxygen species
ER: endoplasmic reticulum
PPAR-α: peroxisome proliferator-activated receptor alpha
IGF-1: insulin-like growth factor-1
HFD: high-fat diet
SD: Sprague-Dawley
CMC: carboxymethyl cellulose
ELISA: enzyme-linked immunosorbent assay
HOMA-IR: homeostasis model assessment of insulin resistance
OS: oxidative stress
DEGs: differentially expressed genes
GO: Gene Ontology
KEGG: Kyoto Encyclopedia of Genes and Genomes
CC: cellular component
MF: molecular function
BP: biological process
PPI: protein-protein interaction
NHANES: National Health and Nutrition Examination Survey
